# From Spider Bite to Fungating Ulcerating Mass: An Aggressive Squamous Cell Carcinoma of the Face

**DOI:** 10.7759/cureus.95768

**Published:** 2025-10-30

**Authors:** Regina J McPherson, Juan Ramon Santos Rivera, Ilya Fonarov, Rodrigo Santoscoy-Valencia, Mehak Sharma

**Affiliations:** 1 Medicine, Florida International University, Herbert Wertheim College of Medicine, Miami, USA; 2 Internal Medicine, Ponce Health Sciences University, Ponce, PRI; 3 Internal Medicine, Jackson Memorial Hospital, Miami, USA; 4 Pathology and Laboratory Medicine, Jackson Memorial Hospital, Miami, USA; 5 Internal Medicine, Florida International University, Herbert Wertheim College of Medicine, Miami, USA

**Keywords:** brown recluse spider bite, facial lesion, hiv-associated malignancies, hiv-related infection, painful skin lesions, squamous cell carcinoma

## Abstract

Necrotic facial lesions can have variable presentations, including presenting as a secondary infection. We present a case of a progressively enlarging necrotic facial lesion in an HIV-positive patient. The patient reported the lesion began five months earlier after a presumed brown recluse spider bite. Despite initial debridement at another hospital, the wound continued to worsen, developing purulent and bloody discharge, escalating pain, and systemic symptoms. On examination, a fungating necrotic mass involving the right face was noted. Imaging suggested possible bony involvement, and biopsy confirmed poorly differentiated squamous cell carcinoma. This case highlights the diagnostic complexity of differentiating between chronic infectious processes and malignancy in immunocompromised patients. The patient’s lack of HIV treatment and unstable living situation contributed to delayed care and advanced disease. Early dermatologic evaluation and biopsy of non-healing lesions are critical, particularly in populations facing medical and social vulnerabilities.

## Introduction

Skin complications are a frequent manifestation of human immunodeficiency virus (HIV), particularly in untreated or advanced disease. Immunosuppression increases the risk of dermatologic conditions, including infections (e.g., folliculitis, candidiasis, herpes simplex, and herpes zoster), inflammatory dermatoses (e.g., seborrheic dermatitis), drug reactions (e.g., Stevens-Johnson syndrome), and neoplasms. Of particular concern are non-melanoma skin cancers, including squamous cell carcinoma (SCC) and basal cell carcinoma (BCC), both of which occur more commonly in HIV-positive individuals [1]. SCC, while less common than BCC, is more aggressive in immunocompromised patients, with higher rates of recurrence and metastasis.

Head and neck SCCs occur at disproportionately higher rates in people with HIV and are often diagnosed at younger ages with worse outcomes. A multi-center cohort of HIV-positive patients with head and neck cancer showed that the median age at diagnosis was 50 years, with most patients being current smokers and having detectable viremia despite highly active antiretroviral therapy (HAART). The study found a strong association between lower CD4 counts and decreased survival, particularly when counts were below 200 cells/μL (reference range: 500-1500 cells/μL) [2].

In rare instances, necrotic or ulcerative skin lesions are initially attributed to arthropod bites, particularly brown recluse spiders (*Loxosceles reclusa*), which are endemic in parts of the United States. Brown recluse envenomation, or loxoscelism, can result in dermonecrosis, blistering, and eschar formation over days. The lesion often evolves from a painful papule to a blue-gray necrotic ulcer with a central crust and surrounding erythema [3]. Systemic symptoms such as fever, hemolysis, or renal injury may also occur, though these are less common. Diagnosis of a brown recluse bite is typically clinical and based on geography, season, and lesion morphology, since the spider is rarely seen [4]. In some cases, particularly among socially vulnerable or immunocompromised individuals, brown recluse bites have been misdiagnosed, delaying recognition of malignancies such as SCC [5].

This diagnostic overlap presents a unique challenge. Some reports have documented lesions initially presumed to be arthropod bites that were later found to be SCC or other serious conditions [6]. Such cases underscore the necessity of biopsy in chronic, non-healing lesions.

## Case presentation

A 62-year-old male with a history of HIV not on HAART, BCC on the arms, hypertension, chronic kidney disease stage 3b due to obstructive uropathy from nephrolithiasis status post right stent placement and left nephrostomy tube placement, and homelessness presented to the emergency room for evaluation of a right facial wound. The patient reported that the lesion had begun five months earlier following a bite from a brown recluse spider. He had been living under a bridge and noted that the facial lesion had progressively worsened, accompanied by increasing pain, which had become nearly unbearable over the past month. Within the last week, the wound began to drain purulent and bloody fluid. He also reported two days of subjective fevers and night sweats. He denied recent weight loss, headache, nausea, vomiting, abdominal pain, chest pain, or shortness of breath.

**Figure 1:**
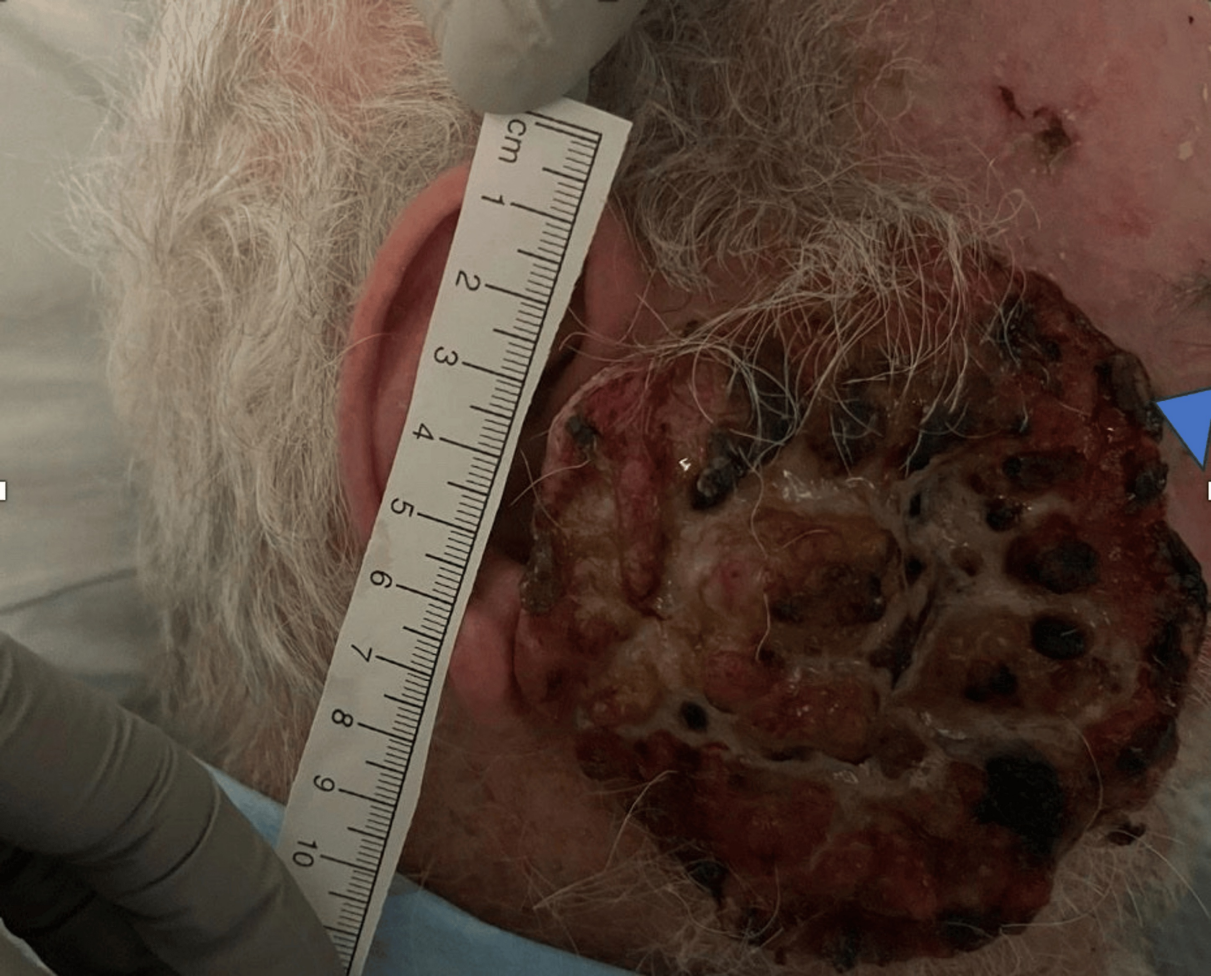
Right facial lesion demonstrating a large, ulcerated, and fungating mass with irregular, heaped borders, measuring approximately 6 cm in diameter. The surface is heterogeneous, with areas of necrosis, hemorrhagic crusting, and purulent exudate. Multiple black eschar-like foci are present across the lesion, along with yellowish slough and granulation tissue. Surrounding skin exhibits sparse terminal hair growth and erythema, suggesting chronic inflammation. The lesion’s raised and lobulated architecture is consistent with advanced cutaneous malignancy, i.e., squamous cell carcinoma with superimposed infection.

The patient had presented to another hospital earlier that same morning, where the wound was reportedly debrided with extensive removal of maggots. He stated that a computed tomography (CT) scan of the brain and the facial bones was performed, showing possible involvement of the zygomatic arch. He was subsequently treated with vancomycin and cefepime, then transferred to our facility for further evaluation by plastic surgery and oncology. Initial vital signs were within normal limits. Physical examination revealed a large, fungating mass on the right side of the face, with a necrotic center and heaped, irregular borders extending from the eyebrows to the ears, consistent with an ulcerated and possibly infected lesion (Figure 1).

**Figure 2:**
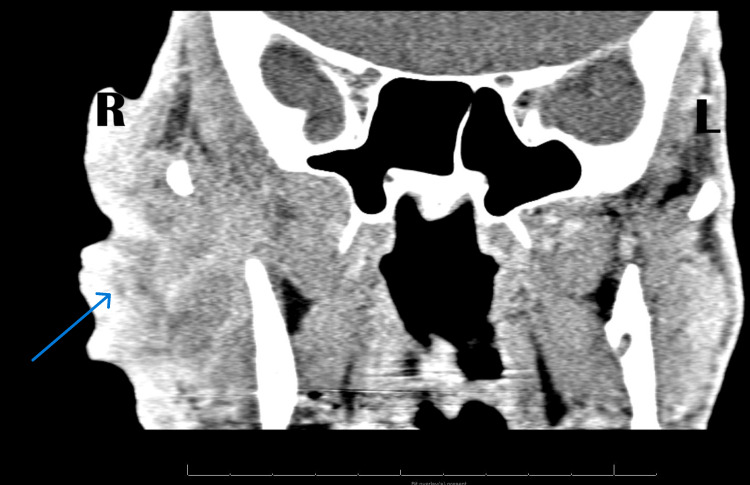
CT of the temporal bone with contrast in axial view showing deep tissue involvement on the right side.

**Figure 3:**
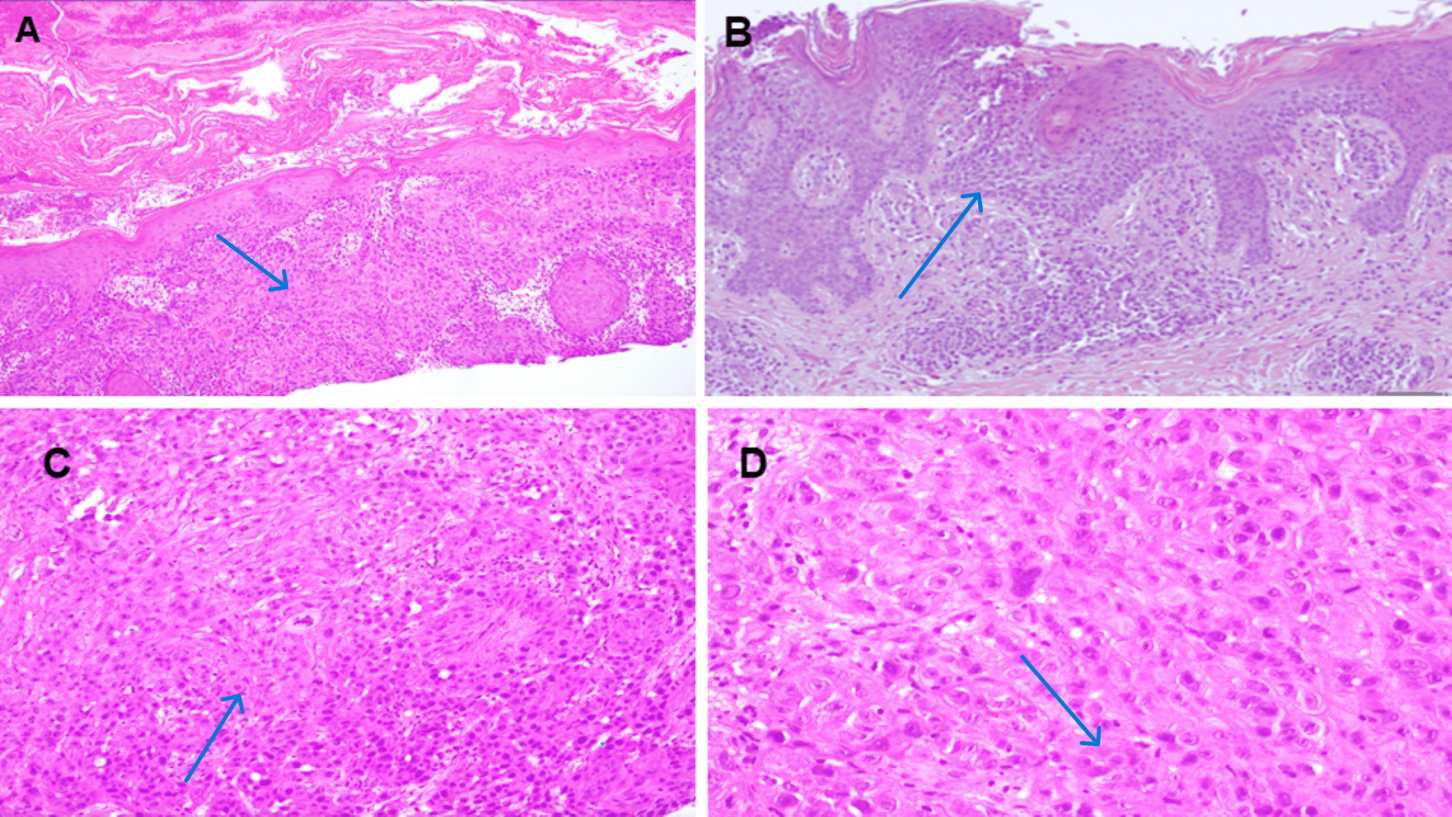
(A) Biopsy showing infiltrating (pink) squamous nests and cords with deep dermal extension emanating from the epidermis. (B) Ulceration and an associated heavy inflammatory cell infiltrate. (C & D) Proliferation of atypical and pleomorphic epithelioid and multinucleated cells with hyperchromatic nuclei, prominent nucleoli, and abundant glassy-appearing eosinophilic cytoplasm. Numerous mitotic figures with atypical forms are present.

There was no palpable lymphadenopathy. Initial laboratory findings were significant for a creatinine level of 2.0 mg/dL (reference range: 0.6 - 1.2 mg/dL), lactic acid of 1.3 mmol/L (reference range: 0.5 - 2.2 mmol/L), and a white blood cell count of 5.0 (5 ×10³/µL) (reference range: 4.5-11 ×10³/µL). His CD4 count was 88 cells/mm^3^ (reference range: 500-1500 cells/mm^3^) (Table 1). The patient was started on vancomycin and cefepime as BCC with superimposed infection was suspected. The infectious diseases team was consulted to guide antibiotic management and further evaluate the untreated HIV. Dermatology was consulted for a biopsy of the lesion. Upon evaluation by infectious diseases, the patient declined HIV workup and initiation of HAART. A biopsy of the facial lesion was performed, and imaging, including a CT of the head, neck, and chest, abdomen/pelvis with contrast, and CT of the temporal bone with and without contrast, was ordered for staging. CT of the temporal bone was significant for a large lobulated skin lesion with deep tissue involvement that extended from the level of the body to the mandible to above the zygoma (Figure 2). CT of the chest was significant for left upper lobe nodules; however, CT of the abdomen/pelvis was negative for metastasis. The biopsy revealed poorly differentiated SCC (Figure 3). Otolaryngology and plastic surgery evaluation for surgical intervention after completion of staging was ordered; however, they agreed that the mass was non-resectable. Oncology evaluation was completed with the ultimate plan for outpatient neoadjuvant immunotherapy treatment with cemiplimab. The patient was seen by a nephrologist, who concluded his creatinine was at baseline.

**Table 1:** Significant laboratory results on admission. BUN: blood urea nitrogen; GFR: glomerular filtration rate; WBC: white blood cell; MCV: mean corpuscular volume; CD4: cluster of differentiation/T helper lymphocytes.

Test	Result	Reference range
Creatinine	2.0 mg/dL	0.6 – 1.2 mg/dL
BUN	41 mg/dL	7 – 20 mg/dL
Albumin	2.8 g/dL	3.5 – 5.0 g/dL
GFR (estimated)	37 mL/min/1.73 m²	>60 mL/min/1.73 m²
WBC	5 ×10³/µL	4.5 – 11 ×10³/µL
Hemoglobin	8.6 g/dL	M: 13.5 – 17.5, F: 12 – 16 g/dL
Hematocrit	30.7%	M: 41 – 53%, F: 36 – 46%
MCV	77.1 fL	80 – 100 fL
Lactic acid	1.3 mmol/L	0.5 – 2.2 mmol/L
CD4 count	88 cells/mm^3^	500 – 1500 cells/mm^3^

## Discussion

This patient presented with a chronic, ulcerated, and necrotic facial lesion in the setting of untreated HIV and homelessness, factors that both expand the differential diagnosis and increase the risk for poor outcomes.

Early diagnostic clarity is critical, as delays can lead to progression, metastasis, and missed therapeutic windows. The differential diagnosis for a non-healing ulcerated facial lesion in this context includes non-melanoma skin cancers (especially SCC and BCC), deep fungal or mycobacterial infections such as *Sporothrix schenckii* or *Mycobacterium marinum*, chronic bacterial infections or abscesses with superimposed necrosis, pyoderma gangrenosum (although rare on the face), necrotic arthropod bites like those from the brown recluse spider, and Kaposi sarcoma, which is classically violaceous but can ulcerate in advanced stages. BCC was considered initially due to the patient’s history and the lesion’s anatomic location. However, BCC typically presents as a slow-growing, pearly papule with central ulceration and rarely causes such profound necrosis or systemic symptoms [1,7]. The lesion’s rapid expansion, foul discharge, and necrotic features were more characteristic of SCC, which was later confirmed histologically. SCC in HIV-positive patients often presents at younger ages, grows aggressively, and has a higher likelihood of recurrence and metastasis, especially when diagnosis is delayed [2,8]. A summary of the main diagnostic considerations in this clinical scenario is provided in Table 2.

**Table 2:** Summary of differential diagnoses for a non-healing ulcerated facial lesion in immunocompromised patients.

Category	Differential Diagnosis	Key Features	References
Malignancy	Squamous cell carcinoma (SCC), basal cell carcinoma (BCC), Kaposi sarcoma	Rapid growth, necrosis, systemic symptoms (SCC); pearly papule with ulceration (BCC); violaceous nodules (Kaposi)	Mohseni et al. (2023) [9]
Infectious (fungal/mycobacterial)	Sporothrix schenckii, Mycobacterium marinum	Chronic lesions, often nodular or ulcerated, are common in immunosuppressed hosts	Barros et al. (2011) [10], Akram et al. (2025) [11]
Infectious (bacterial)	Chronic bacterial abscesses with superimposed necrosis	Purulent drainage, foul odor, localized erythema, and tenderness	Chandler et al. (2024) [12]
Inflammatory	Pyoderma gangrenosum	Painful ulcers with undermined borders, associated with systemic diseases, and rare on the face	Maronese et al. (2022) [13]
Toxic/envenomation	Brown recluse spider bite (loxoscelism)	Central necrosis with surrounding erythema and edema; systemic symptoms in severe cases	Abdelazeem et al. (2021) [3]
Viral-related tumors	Kaposi sarcoma (human herpesvirus 8 (HHV-8) associated)	Violaceous plaques or nodules can ulcerate in late stages, common in advanced HIV	Mohseni et al. (2023) [9]

The patient’s reported history of a brown recluse spider bite added further diagnostic complexity. While loxoscelism can produce necrotic skin ulcers, this diagnosis is often made clinically and is frequently over-attributed in endemic regions. Brown recluse bites typically evolve over days into centrally necrotic lesions with surrounding erythema and edema, occasionally accompanied by systemic symptoms such as fever and malaise. However, in the absence of observed envenomation, tissue biopsy is essential when lesions worsen or persist beyond the typical healing window of two to six weeks [3,4]. Moreover, exaggerated arthropod bite reactions and misattributed necrosis have been well-documented as mimics of SCC and other malignancies, particularly in patients with HIV [5,6].

Management of chronic ulcerative lesions in immunocompromised patients requires a multidisciplinary approach. Early biopsy is critical for diagnosis and should not be delayed when malignancy is suspected. Empiric broad-spectrum antibiotics may be initiated when superimposed infection is likely, but persistent or enlarging lesions should prompt early dermatologic and oncologic consultation. Imaging is essential when there is suspicion of local invasion or bony involvement, as was the case here. HIV management should be concurrently addressed, ideally with antiretroviral therapy (ART) initiation to support immune reconstitution, although in this case, the patient declined.

Importantly, this case also highlights the central role of social determinants of health. These factors not only contributed to the advanced stage of his malignancy but also reduced his eligibility for potentially curative therapy. The literature shows that HIV-positive patients with head and neck SCC have significantly worse outcomes when CD4 counts are low, viral load is uncontrolled, and tobacco use is present, all factors frequently associated with unstable living conditions [2]. Without addressing these upstream barriers, termed the “causes of the causes” in public health literature, clinical interventions alone may fall short [14]. Vigilance is particularly warranted when the clinical picture overlaps with common mimics like arthropod bites, as delay in diagnosis can have life-threatening consequences.

## Conclusions

This case highlights the diagnostic and management challenges of chronic facial ulcers in immunocompromised individuals, particularly when clinical features mimic infectious processes such as spider bites. In patients with untreated HIV, rapidly progressing skin lesions should prompt early biopsy to rule out malignancy, especially SCC, which is more aggressive in this population. Delayed diagnosis in this case was further compounded by homelessness, emphasizing the need to address social determinants of health alongside medical care. A multidisciplinary approach that integrates timely dermatologic evaluation, oncologic planning, and social support is essential to improving outcomes in vulnerable populations.
